# A p-Median approach for predicting drug response in tumour cells

**DOI:** 10.1186/s12859-014-0353-7

**Published:** 2014-10-29

**Authors:** Elisabetta Fersini, Enza Messina, Francesco Archetti

**Affiliations:** Department of Informatics, Systems and Communication, University of Milano-Bicocca, Viale Sarca, 336 Milan, Italy; Consorzio Milano Ricerche, Viale Cozzi, 53 Milan, Italy

**Keywords:** p-Median clustering, Bayesian networks, Drug response prediction

## Abstract

**Background:**

The complexity of biological data related to the genetic origins of tumour cells, originates significant challenges to glean valuable knowledge that can be used to predict therapeutic responses. In order to discover a link between gene expression profiles and drug responses, a computational framework based on Consensus p-Median clustering is proposed. The main goal is to simultaneously predict (in silico) anticancer responses by extracting common patterns among tumour cell lines, selecting genes that could potentially explain the therapy outcome and finally learning a probabilistic model able to predict the therapeutic responses.

**Results:**

The experimental investigation performed on the NCI60 dataset highlights three main findings: (1) Consensus p-Median is able to create groups of cell lines that are highly correlated both in terms of gene expression and drug response; (2) from a biological point of view, the proposed approach enables the selection of genes that are strongly involved in several cancer processes; (3) the final prediction of drug responses, built upon Consensus p-Median and the selected genes, represents a promising step for predicting potential useful drugs.

**Conclusion:**

The proposed learning framework represents a promising approach predicting drug response in tumour cells.

**Electronic supplementary material:**

The online version of this article (doi:10.1186/s12859-014-0353-7) contains supplementary material, which is available to authorized users.

## Background

Cancer is a disease treated with various strategies depending on the type of cancer and the stage of the disease. Generally, therapeutic agents are selected according to the specific cancer type and patient population, based on the effectiveness in large-population studies [1,2]. Now, with the advances of the genomic era, a massive amount of high-throughput data has been made available for understanding the cancer system biology. The public available datasets composed of genomic data and drug responses offer the opportunity to reveal valuable knowledge about the hidden relationships between gene expression and drug activity of tumor cells, pointing out the conditions that bring a patient to be more responsive than others to a given therapeutic agent. Although data collection provides the baseline to enable a better understanding of cancer mechanisms, data integration and interpretation is still an open issue. Mathematical and statistical models of complex biological systems play a fundamental role in system biology, and in particular in cancer related issues. They can be exploited for exploratory purposes, to validate hypothesis and make predictions about quantities that are difficult or impossible to be measured *in vivo*.

In the last decade, several studies have been conducted to develop platforms on which cancer high-throughput computational analysis can be performed. Much of these computational approaches are targeted at predicting the drug sensitivity/resistance by means of statistical inference and regression methods able to take into account genomic information of hundreds of genes for determining a specific drug response [3-5]. However, the massive availability of chemical compounds as potential cancer therapies has opened to the investigation of *in silico* therapy response prediction which requires more sophisticated computational models and methods to optimize the experimental design of cell-drug screenings.

A first attempt of gene-drug integrative analysis was presented in [6] where, thanks to a hierarchical clustering algorithm, several investigations have been performed: (1) cell-to-cell correlation on the basis of gene expression and drug activity profiles, (2) relationships between drug activity patterns and mechanisms of action, (3) gene-drug correlation on the basis of gene expression and drug activity profiles. In subsequent investigations [7,8], the triangle gene expression profiles, drug responses and cancer types has been explored by integrating unsupervised and supervised machine learning algorithms. The clustering approach based on Soft Topographic Vector Quantization (STVQ) [9] has shown that gene expression profiles are more related to the cancer type than to the drug activity patterns, while thanks to the structure learning of Bayesian Networks (BN) some biologically meaningful relationships among gene expression levels, drug activities and cancer types have been confirmed and in some cases revealed. More recent works [10-13] are targeted at integrating explorative approaches with predictive paradigm towards a computational gene-drug screening. While [10,11] and [12] are based on non-deterministic clustering approaches (k-Means, STVQ and Genetic Programming) for identifying relevant genes involved in cancer mechanisms and predictive of drug response, [13] introduces a framework based global optimization to cancel the randomness, and therefore the variance, of stochastic clustering results when predicting a therapy outcome.

The results of the above quoted papers and of a wide set of related approaches highlight several interesting considerations:

- The explorative analysis performed through clustering approaches reveals that the tissue of origin is more related to the gene expression profile than the drug activity patterns. This suggests that the genomic information of a cell line plays a fundamental role, independently of the organ of origin, to understand anticancer therapy responses. This idea has been supported by the fact that several cell lines with a relatively high expression level of those genes regulating multi-drug resistance have been clustered in the same group. This indicates that chemoresponse mechanisms are distributed across different tissues in the panel and that it should be possible to link drug responses to gene expression profiles.
- In order to cancel the variability of results of stochastic clustering and to guarantee the convergence to a global minimum, we need to address the clustering problem by exact approaches able to find globally optimal solutions.
- Computational approaches based on Bayesian Networks reveal interesting relationships among subsets of genes and drugs. The potential of Bayesian Networks encourages us to exploit this probabilistic model not only for deductive purposes, but also for prediction issues.

In order to achieve the final goal of simultaneously predicting the drug response of several compounds given a patient genomic profile, we propose a computational framework based on the following assumption: groups of cell lines homogeneous in terms of both gene expression profile and drug activity should be characterized by a subset of genes that explains the drug responses. To this purpose a three-folds analysis has been investigated: p-Median problem formulations to create clusters of homogeneous cell lines, Feature Selection Policies to select relevant genes and finally Bayesian Networks to predict drug responses of tumour cell lines. Computational results show that the proposed Consensus p-Median, combined with gene selection and BN inference engine, yields homogeneous clusters while guaranteeing good predictive power for inferring drug responses for a new cell line. This is also confirmed by the biological evaluation performed on the selected genes: according to the existing literature the set of genes used to train the BNs, which has been selected by using the groups of cell lines obtained by the proposed Consensus p-Median, has shown to be biologically relevant from an oncological point of view.

## Methods

### Problem formulation

The problem of simultaneously predicting the response of several therapeutic compounds given the patient genomic profile is addressed by a computational framework composed of three main building blocks:

1. The creation of homogeneous groups of tumor cell lines by means of p-Median formulations. In particular, a novel Consensus p-Median formulation is proposed and compared with traditional state of the art approaches, i.e. k-Means [14], STVQ [9] and Relational k-Means [11] and Probabilistic D-Clustering [15].
2. The selection of relevant genes able to predict the response of hundreds of drugs. We explore the potential of the solutions determined by solving the above mentioned p-Median problem formulation for identifying a subset of genes that characterizes each cluster, i.e. those subsets of genes that could be responsible of drug responses. To accomplish this task two main feature selection policies have been investigated, i.e. Information Gain [16] and Correlation-based Feature Subset Evaluation (CFS) [17].
3. The simultaneous prediction of different drug responses by exploiting the potential of Bayesian Networks [18]. Establishing a straightforward dependency structure of the Bayesian Network, we explore the ability of the selected genes to predict a panel of drug responses given the genomic profiles of patients.

The proposed computational framework exploits the well known dataset provided by the U.S. National Cancer Institute. The dataset consists of 60 cell lines from 9 kinds of cancers, all extracted from human patients, where the tumors considered in the panel derive from colorectal, renal, ovarian, breast, prostate, lung and central nervous system as well as leukemia and melanoma cancer tissues.

For the cell lines in the panel, both transcript profiling and chemosensitivity patterns have been considered. In the following we will consider two datasets stemmed from the original one: (a) Scherf et al. based on cDNA arrays and (b) Liu et al. based on microRNA arrays. In both cases the dataset is defined as a set *Ω* of all cell lines *x*_*i*_, with *i*={1,…60}, into the real vector space $\mathbb {R}^{m+n}$:

(1) $$\begin{array}{@{}rcl@{}} \Omega = \left\{x_{i}| x_{i}=\left({x_{i}^{G}}, {x_{i}^{D}}\right), x^{G}\in \mathbb{R}^{m}, {x_{i}^{D}}\in \mathbb{R}^{n}\right\} \end{array} $$

where ${x_{i}^{G}}$ represents the transcript expression level as a vector into the space $\mathbb {R}^{m}$ and ${x_{i}^{D}}$ denotes the drug response as a vector into the space $\mathbb {R}^{n}$.

#### Sherf dataset: cDNA arrays and DTP-tested chemical compounds

The Sherf dataset, originally presented in [6], denotes the gene expression profile ${x_{i}^{G}}$ by using the cDNA microarray technology and the drug response ${x_{i}^{D}}$ by assessing the grown inhibition activities (*GI*_50_) after 48 hours of drug treatment through Sulphorhodamine B. We can consequently define *Ω*^*G*^ and *Ω*^*D*^ as the set of cell lines represented through their gene expression profiles and their drug activity responses respectively:

(2) $$\begin{array}{*{20}l} \Omega^{G} = \left\{{x_{i}^{G}}| x_{i}=\left({x_{i}^{G}}, {x_{i}^{D}}\right), x\in \Omega\right\} \end{array} $$

(3) $$\begin{array}{*{20}l} \Omega^{D} = \left\{{x_{i}^{D}}| x_{i}=\left({x_{i}^{G}}, {x_{i}^{D}}\right), x\in \Omega\right\} \end{array} $$

According to the Sherf representation $\mathbb {R}^{m}$, with *m*= 1375, includes genes selected from the original NCI60 dataset (characterized by 9073 genes) having 5 or fewer missing values and showing strong pattern of variation among the 60 cell lines (more than 3 measurements must have red-green intensity ratios >2.6 or <0.38). The space $\mathbb {R}^{n}$, with *n*=1400, includes drugs contained into the original dataset, where each compound has been tested one at time and independently. Considering that among 1375 genes and 1400 drugs missing values were still present, they have been replaced by the average gene expression value (or the average drug activity) over the 60 cell lines. The gene expression profiles and drug activity response for Sherf dataset are available for download as Additional file 1: Sherf gene expression data and Additional file 2: Sherf drug activity data.

#### Liu dataset: microRNA arrays and drugs with known mechanism of action

MicroRNAs (miRNA in the following) are a group of short noncoding RNAs that regulate gene expression at the post-transcriptional level. They are involved in many biological processes, including development, differentiation, apoptosis, and carcinogenesis. Because miRNAs may play a role in the initiation and progression of cancer, they comprise a novel class of promising diagnostic and prognostic molecular markers and potential drug targets. In order to achieve our goal by exploiting the miRNA data, we considered the dataset presented in [19]. This dataset leads us to represent the sets *Ω*^*G*^ and *Ω*^*D*^ by means of 422 miRNA expression profile and 118 *GI*_50_ responses related to drugs with known mechanism of action. The same selection criterion applied on Sherf dataset has been exploited for Liu dataset. Concerning the miRNA expressions, in this dataset there are no missing values and more than 3 experiments have red-green intensity ratios >2.6 or <0.38, implying no selection of miRNA and therefore a space $\mathbb {R}^{m=422}$. Regarding the drug space, $\mathbb {R}^{n=118}$ is characterized by the presence of missing values. As well as for Sherf dataset, they have been replaced by the average drug activity over the 60 cell lines. The miRNA expression profiles and drug activity response for Liu dataset are available for download as Additional file 3: Liu miRNA expression data and Additional file 4: Liu drug activity data.

### Cluster analysis

Cluster analysis is aimed at discovering embedded patterns into a given dataset. From a high level point of view cluster analysis consists of partitioning a set of patterns into subsets (clusters) based on similarity, i.e. a cluster has to contain similar patterns and dissimilar patterns have to be in different clusters. This could be accomplished by partitioning data points into a pre-specified number of clusters through the optimization of a cost function related to a similarity/dissimilarity measure between data points.

An important step in any clustering algorithm is to select a distance measure, which will determine how the similarity/dissimilarity of two data points is calculated. In order to perform a cluster analysis we chose one of the most used distance measures [20] based on Pearson Correlation (*corr*):

(4) $$\begin{array}{@{}rcl@{}} d\left(x_{i},x_{j}\right) = 1-corr\left(x_{i},x_{j}\right) \end{array} $$

where *x*_*i*_ and *x*_*j*_ represent two cell lines and *corr*(*x*_*i*_,*x*_*j*_) denotes the Pearson Correlation coefficient between *x*_*i*_ and *x*_*j*_. Thanks to the distance measure denoted by equation (4), cell lines having high distance due to anti-correlated genes/drugs are likely placed in different clusters, while cell lines characterized by a small gap are expected to be clustered together. The adoption of a correlation-based metric instead of the Euclidean distance is motivated by its sensitivity with respect to magnitude: Euclidean distance is sensitive to scaling and differences in average expression level, whereas correlation is not.

In the following we present three different clustering approaches based on p-Median formulation: traditional p-Median, probabilistic d-Clustering and Consensus p-Median.

#### Traditional p-Median

The p-Median problem was originally designed for facility location planning [21], where the location of “*p*-facilities” relative to a set of “customers” has been formulated such that the sum of the shortest demand weighted distance between “customers” and “facilities” is minimized. In our investigation, the p-Median problem has been formulated as an assignment problem for creating groups of cell lines by using a “flat” representation of data, i.e. by representing each cell line as a vector in $\mathbb {R}^{m+n}$. Given a cell line *x*_*i*_∈*Ω* and *K* desired clusters, the clustering problem consists in assigning each *x*_*i*_ to a cluster center *x*_*j*_, such that the intra-cluster distance is minimized and the inter-cluster distance is maximized.

Let *Z* be a matrix of dimension |*Ω*|×|*Ω*|, as:

(5) $$ \begin{array}{ll} z_{ij}= \left\{ \begin{array}{ll} 1 & \textrm{if \(x_{i}\) \textrm{is associate to the cluster center} \(x_{j}\)}\\ 0 & \text{otherwise} \end{array}\right. \end{array}  $$

where *z*_*ij*_ represents the assignment variable that indicates whether a cell line *x*_*i*_ is assigned to a cluster center *x*_*j*_. Note that the matrix *Z* has dimension |*Ω*|×|*Ω*| because each entry *z*_*ij*_ denotes the potential association of a cell line *x*_*i*_ to any of the points *x*_*j*_ in *Ω* (where *x*_*j*_ can be a cluster center or not).

The clustering problem can be formulated as follows:

(6) $$ \min F= \displaystyle\sum\limits_{i=1}^{\vert\Omega\vert} \displaystyle\sum\limits_{j=1}^{\vert\Omega\vert} z_{ij} d\left(x_{i},x_{j}\right)  $$

s.t:

(7) $$ \displaystyle\sum\limits_{j=1}^{\vert\Omega\vert} z_{ij} = 1 \hspace{1cm} \forall i \in \lbrace1,2, \ldots,\vert\Omega\vert\rbrace  $$

(8) $$ \displaystyle\sum\limits_{j=1}^{\vert\Omega\vert}z_{jj} =K  $$

(9) $$ z_{ij} - z_{jj} \leq 0 \hspace{1cm} \forall i,j \in \lbrace1,2, \ldots,\vert\Omega\vert\rbrace  $$

(10) $$ z_{ij} = \{0,1\} \hspace{1cm} \forall i,j \in \lbrace1,2, \ldots,\vert\Omega\vert\rbrace  $$

According to this formulation, the objective function in equation (6) denotes a combinatorial optimization problem whose objective is to minimize the distance between all data points belonging to the same cluster through the identification of optimal cluster centers *x*_*j*_∈*Ω*. Constraint (7) ensures that each cell line *x*_*i*_ is assigned to only one cluster, constraint (8) guarantees that there will be exactly *K* clusters and constraint (9) ensures that if *x*_*i*_ is assigned to *x*_*j*_ then *x*_*j*_ is a cluster center and therefore a median. The last constraint (10) guarantees integrality.

For seek of clarity, the above mentioned p-Median is a *mathematical programming formulation* (also known as generalized Fermat-Weber problem formulation) for uncapacitated facility location problems. The objective of this formulation is to minimize the sum of the distances from all data points *x*_*i*_ to their respective cluster centers (geometric medians). In this paper the p-Median problem is solved deterministically^a^ by means of a canonical “branch and cut” algorithm [22]. The solution of the p-Median problem finds out not only the cluster assignments, but also the geometric medians as cluster representatives.

p-Median must not be confused with approaches like k-Means [14], k-Medoids [23] and k-Medians [24], which represent *heuristic algorithms* for approximating the above mentioned objective function. While k-Means computes a cluster representative (centroid) as mean vector of all points belonging to a cluster, k-Medoids and k-Medians select respectively *k* of the |*Ω*| data points as medoids (whose average distance to all the objects in the cluster is minimal) and medians (combination of multiple instances). On the contrary, a branch-and-cut algorithm on a p-Median formulation determines the set of *p* data points that minimize the sum of weighted distances to any points of the dataset and consequently finds out the cluster assignment for each data point. The geometric medians determined by solving the p-Median problem do not coincide neither with the centroids, medians or medoids (the only exceptions are for the 1-dimensional case, where the geometric median coincides with the median and when in k-Medoids the medoids are selected as median objects instead of computed as combination of multiple instances). In our investigation, the solution of the p-Median problem formulations are ensured to be the global optimum, while the ones originated by the heuristic approaches can correspond to local optimum among all possible solutions.

#### Probabilistic D-Clustering

The assignment problem presented above assumes to create *K* mutually exclusive clusters of cell lines, with similar profiles of gene expression and drug response. The crisp formulation can be relaxed by modelling probabilistic (or soft) assignments (with cluster membership probabilities), leading to a probabilistic p-Median named Probabilistic D-Clustering [15].

The formulation reported in (6)-(10) can be therefore approximated by the following minimization problem:

(11) $$ \min F_{p}= \displaystyle\sum\limits_{k=1}^{K} \displaystyle\sum\limits_{j=1}^{\vert\Omega\vert} {p_{k}^{2}}\left(x_{i}\right)d\left(x_{i},c_{k}\right)  $$

s.t:

(12) $$ \displaystyle\sum\limits_{k=1}^{K} p_{ik}=1 \hspace{1cm} \forall i \in \lbrace1,2, \ldots,\vert\Omega\vert\rbrace  $$

(13) $$  p_{ik}\geq 0 \hspace{.8cm} \forall i \in \lbrace 1,2, \ldots,\vert\Omega\vert\rbrace \hspace{.9cm} \forall k \in \lbrace1,2, \ldots,K\rbrace  $$

where the decision variables *c*_*k*_ and *p*_*k*_(*x*_*i*_) denote the cluster centers *c*_*k*_ and the probability of assigning the cell line *x*_*i*_ to the cluster *c*_*k*_ respectively. Each cell line can be finally assigned to the cluster center with the highest probability.

It can be easily noted that the formulation of Probabilistic D-Clustering is a further generalization of the p-Median (Fermat-Weber) problem, slightly different from the ones presented in equation (6)-(10) but still belonging to the combinatorial optimization. While for Traditional p-Median the creation of *K* clusters is forced by the constraint (8), in Probabilistic D-Clustering the generation of *K* clusters is driven by the objective function.

A natural working principle to solve (11)-(13) is to fix one set of variables and minimize the objective function with respect to the other set of variables, then fix the other set and minimize again, until convergence is achieved. An iterative method has been recently proposed to solve the problem, leading to a generalized Weiszfeld method [25], where centers and probabilities are sequentially updated. The iterative method alternates between:

- Step 1: Probabilities Update. Given the centers *c*_*k*_ and the distance between each cell line *x*_*i*_ and *c*_*k*_, the probabilities that *x*_*i*_ is assigned to the cluster *k* can be estimated as:
  (14) $$ p_{k}\left(x_{i}\right)=\frac{\prod\limits_{j \neq k}d\left(x_{i},c_{j}\right)}{\sum\limits_{l=1}^{K}\prod\limits_{m \neq l}d\left(x_{i},c_{m}\right)}  $$
  Given the clusters, their centers, and the distances of data points from these centers, the probability of cluster membership at any point is assumed inversely proportional to the distance from (the center of) the cluster.
- Step 2: Centers Update. Given the probabilities *p*_*k*_(*x*_*i*_), the centers $c_{k}^{+}$ can be updated according to the current cluster distribution as:
  (15) $$ c_{k}^{+}=\frac{\sum\limits_{i=1}^{|\Omega|} \mu_{k}\left(x_{i}\right)x_{i}}{\sum\limits_{j=1}^{| \Omega |} \mu_{k}\left(x_{j}\right)}  $$
  where
  (16) $$ \mu_{k}\left(x_{i}\right)=\frac{p_{k}\left({x_{i}^{2}}\right)}{d\left(x_{i},c_{k}\right)}  $$
  The centers are updated as convex combinations of these points, with weights determined by the working principle.

The iterative process stops when the centers stabilize, i.e. when

(17) $$ \sum\limits_{k=1}^{|K|} \| c_{k}^{+} - c_{k} \| < \epsilon  $$

originating a clustering of cell lines in the space $\mathbb {R}^{m+n}$. The optimal clustering solution can be determined (see ref. [15]) by verifying the optimality of centers and assignments through the dual problem corresponding to the primal reported in Eq. (11)-(13).

#### Consensus P-Median

The cluster analysis of the NCI60 dataset relates to a set of objects (cell lines) that need to be grouped taking into account multiple sources (gene expression profiles and drug activity patters). Most of the multi-source clustering approaches follow one of the following paradigms: (a) clustering each data source separately to then ad-hoc integrate the separate clustering solutions [26,27] or (b) combining all data sources to determine a single “joint” clustering [28,29] as Traditional p-Median and Probabilistic D-Clustering. The first kind of approaches is characterized by an independent analysis: while they take advantage of modeling source-specific features, they are not able to capture inter-source associations. On the other side, the second type of approaches is based on a joint analysis that is able to exploit shared structure among data sources, but disregarding the heterogeneity of the data and taking no account important features that are specific to each data source. More flexible methods allow for separate but dependent source clusterings [30,31].

The characteristics of these more flexible approaches, along with the remarks highlighted in Background section, led us to define a Consensus p-Median formulation based on two steps: the first is aimed at determining groups of cell lines into the gene (or drug) space, whereas the second one determines the clusters of cell lines into the drug (or gene) space, while constraining the optimal solution in order to take into account the assignment of the first step. This approach aims at finding a trade-off between gene expression and drug response profiles, by defining a sequence of two integer linear programming formulations. While the problem at the first step can be formulated as a traditional p-Median (in one of the two spaces, i.e. either gene or drug space), the second step leads to the definition of the following Consensus p-Median formulation:

(18) $$ \min F_{r} = \displaystyle\sum\limits_{i=1}^{\vert\Omega\vert}\displaystyle\sum\limits_{j=1}^{\vert\Omega\vert} z_{ij} d^{(1)}\left(x_{i},x_{j}\right)  $$

s.t:

(19) $$ \displaystyle\sum\limits_{i=1}^{\vert\Omega\vert} \displaystyle\sum\limits_{j=1}^{\vert\Omega\vert} z_{ij} d^{(2)}\left(x_{i},x_{j}\right)\leq \mu \cdot \displaystyle\sum\limits_{i=1}^{\vert\Omega\vert} \displaystyle\sum\limits_{j=1}^{\vert\Omega\vert} z_{ij}^{*} d^{(2)}\left(x_{i},x_{j}\right)  $$

(20) $$ \displaystyle\sum\limits_{j=1}^{\vert\Omega\vert}z_{ij} = 1 \hspace{1cm} \forall i \in \lbrace1,2, \ldots,\vert\Omega\vert\rbrace  $$

(21) $$ \displaystyle\sum\limits_{i=1}^{\vert\Omega\vert} z_{ii} =K  $$

(22) $$ z_{ij} - z_{jj} \leq 0 \hspace{1cm} \forall i,j \in \lbrace1,2, \ldots,\vert\Omega\vert\rbrace  $$

(23) $$ z_{ij} = \{0,1\} \hspace{1cm} \forall i,j \in \lbrace1,2, \ldots,\vert\Omega\vert\rbrace  $$

where $z_{\textit {ij}}^{*}$ denotes the solution of problem (6)-(9). This problem formulation consists in assigning each cell line *x*_*i*_ to a given cluster according to a distance measure computed in one space *d*^(1)^(for example the gene space). The constraints denoted by equations (20)-(23) have the same role as in the traditional p-Median formulation, while equation (19) provides a constraint about the cluster assignment by taking into account the cluster placement occurred during the first step. In particular, this constraint avoids the clustering solution of the Consensus p-Median to diverge, according to a value *μ* and to the distance measure *d*^(2)^≠*d*^(1)^, from the solution found at the first step. The parameter *μ* tunes the effect of the solution that optimizes *F*, i.e. $\displaystyle \sum \limits _{i=1}^{\vert \Omega \vert } \displaystyle \sum \limits _{j=1}^{\vert \Omega \vert } z_{\textit {ij}}^{*} d_{\textit {ij}}^{(2)}$. The parameter *μ* ranges between the lower bound *μ*=1.0 and an upper bound *μ*^∗^. *μ*=1 implies that the solution of the Consensus p-Median will generate the same assignment as the traditional p-Median solved at the first step. Increasing values of *μ* cause a decreasing effect of optimal assignment $z_{\textit {ij}}^{*}$ coming from the first phase (*μ* can be updated incrementally until the convergence criterion is satisfied, i.e. the solution of the Consensus p-Median doesn’t change for increasing values of *μ*). Note that for *μ*<1.0 no feasible solution exists.



The pseudo-code reported in Algorithm ?? summarizes the iterative process for solving the Consensus p-Median until the value of *μ*^∗^ is found, i.e. until constraint (19) becomes redundant. For the sake of simplicity, we will denote with *Consensus p-Median (g-d)* the approach where at step 2 the set *Ω*^*G*^ is used and at steps 3 and 8 the set *Ω*^*D*^ is exploited. On the other hand, we will denote with *Consensus p-Median (d-g)* the approach where *Ω*^*D*^ is exploited at step 2, while at step 3 and 8 the set *Ω*^*G*^ is used.

### Feature selection

The clusters that can be generated by the above mentioned approaches represent sets of cell lines that show a similar response to anti-cancer therapy also taking into account genomic information. This enables a feature selection activity that allows us to to identify the subset of genes that could possibly regulate the cell response behavior. To compactly characterize the obtained clusters, we attempt to select a subset of genes that best represents the cell lines membership. In order to validate the hypothesis that the obtained groups of cell lines embed useful information for helping the pharmacology of cancer, we applied two feature selection techniques known as Information Gain and Correlation-based Feature Subset Evaluation.

#### Information gain

In order to determine the most relevant genes that characterize a cluster and therefore that can be responsible of drug response for the cell lines belonging to that cluster, a feature selection based on Information Gain has been applied. Information Gain measures the decrease in entropy when the feature is given vs absent. According to this measure a “good” feature can contribute, independently of any other feature, to reduce the uncertainty of each clusters given the attribute values. Formally, given a cluster attribute *C* representing the obtained clusters and a gene attribute *A*, denoting the expression level of a given gene, the Information Gain (IG) is computed as follows:

(24) $$ IG(C,A)=H(C)-H(C|A)  $$

where

(25) $$ H(C)=\sum_{k=1}^{K}P(c_{k}) \log P\left(\frac{1}{c_{k}}\right)  $$

(26) $$ H(C|A)=\sum_{k=1}^{K}\sum_{t=1}^{T}P\left(c_{k},a_{t}\right) \log \frac{P\left(a_{t}\right)}{P\left(c_{k},a_{t}\right)}  $$

We can therefore consider equation (26) as a measure of dependency between the density of variable *a*_*t*_ (gene) and the distribution of the target *c*_*k*_ (cluster).To compute the entropy in equation (26), the *T* nominal expression values need to be represented as discrete quantities. In order to discretize genes as up-, down- and normo- regulated, a double filtering approach has been applied.

**Figure 1:**
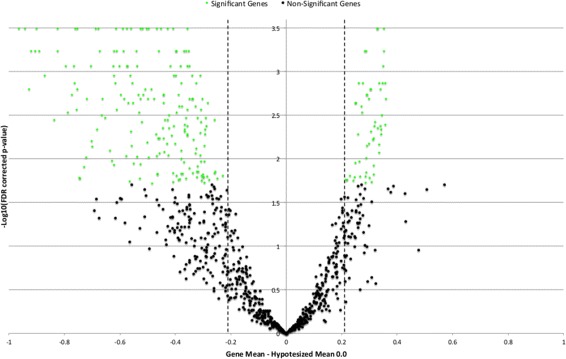
**Volcano plot.** Volcano plot of *l* *o* *g*_2_ fold-change (x-axis) versus −*l* *o* *g*_10_ FDR-corrected p-value (y-axis, representing the probability that the gene is differentially expressed). Genes with log fold change above −*l* *o* *g*_2_(0.86)=0.2175 are up-regulated, while genes with log-fold change below *l* *o* *g*_2_(0.86)=−0.2175 are considered as down regulated.

In order to discretize genes as up-, down- and normo- regulated, a double filtering approach has been applied. In particular, genes that are differentially expressed have been identified by applying FDR corrected p-value test [32], with the requirement that the rate of false significant genes should not exceed 5% with a confidence of 99%. Once non-significant genes have been identified, a mean difference cut-off (on the log fold changes) has been applied to discriminate between up- and down-regulated genes among the significant ones. The mean difference cut-off *β* corresponds to the minimum absolute value of expression such that a gene is not considered as non-significant. With FDR corrected p-value, the mean difference cut-off corresponds once more to *β*=0.86 (both Sherf and Liu datasets show a cut-off *β*=0.86). According to the double filtering approach, genes with a fold change >+0.86 are considered as up-regulated, while genes with a fold change <−0.86 are considered as down-regulated. Gene expression values into the interval [ −0.86,0.86] are identified as normo-regulated. The discretization threshold can be easily grasped by looking at the volcano plot reported in Figure 1.

Once genes have been discretized, the value of *IG*(*C*,*A*) for each attribute can be computed allowing genes to be ranked accordingly*b*. The top 10 genes have been selected as the most representative to train the predictive model described subsequently.

#### Correlation-based feature subset evaluation

An alternative feature selection method, able to evaluate the contribution of each gene, is the Correlation-based Feature Subset Evaluation (CFS). This approach assumes that good feature subsets contain features highly correlated with the cluster attribute, while yet uncorrelated with each other. The selection algorithm, which takes as input the genes discretized according to the FDR corrected p-value test introduced above, is a heuristic that evaluates the merit of a subset of features, taking into account the usefulness of individual features for predicting the class label (cluster assignment) along with the level of intercorrelation among them. The merit of a subset *S* composed of *g* features can be estimated as:

(27) $$ Merit(S)=\frac{g r_{bf}}{\sqrt{g(g+g-1)r_{ff}}}  $$

where *r*_*bf*_ is the mean feature-cluster correlation, and *r*_*ff*_ is the average feature-feature intercorrelation. The numerator can be viewed as an indication of how predictive of the cluster a set of features are, while the denominator of how much redundancy there is among the features^b^. More details about CFS can be found in [17].

### Prediction

The results obtained in the previous steps allow us to train a predictive model able to infer, for a new cancer patient, the multiple drug responses by using his/her gene expression profile of selected genes. One way of deriving a predictive model is to estimate a joint distribution for the set *Q* of features that characterize the dataset. The joint distribution for a sample *x*_*i*_∈*Ω*, where $x_{i} = \left \{{x_{i}^{1}}, {x_{i}^{2}},..,x_{i}^{|Q|}\right \}$, can estimated over the feature space as:

(28) $$\begin{array}{@{}rcl@{}} P\left(x_{i}\right) = P\left({x_{i}^{1}}, {x_{i}^{2}},..,x_{i}^{|Q|}\right) \end{array} $$

A complete joint probability distribution over a set of random variables must specify a probability value for each of the possible set instantiation. For example, if we consider to specify an arbitrary joint distribution *P*(*X*^1^,*X*^2^,..,*X*^|*Q*|^) for |*Q*| dichotomous variables, a table with 2^|*Q*|^ entries is required. This complexity makes an infeasible probability model for any domain of realistic size. A possible solution that tries to overcome this problem is represented by Bayesian Networks [18]. The key component, that reduces the probability model complexity, is the assumption that each variable is directly influenced by only few others.

This assumption is captured graphically by the dependency structure: a probability distribution is encoded by a directed acyclic graph whose nodes represent random variables and edges denote direct dependencies. Formally, a Bayesian Network asserts that each node (random variable) is conditional independent of its non-descendants given its parents. This conditionally independence assumption allows us to represent concisely the joint probability distribution over the random variables.

If we consider a distribution over |*Q*| features, which can be arbitrarily ordered as *X*^1^,*X*^2^,..,*X*^|*Q*|^, it can be decomposed as the product of |*Q*| conditional distributions:

(29) $$\begin{array}{@{}rcl@{}} P\left({x_{i}^{1}}, {x_{i}^{2}},..,x_{i}^{|Q|}\right)=\prod\limits_{s}P\left({x_{i}^{s}}|{x_{i}^{1}},..,x_{i}^{s-1}\right) \end{array} $$

Instead of specifying the probability of *X*^*s*^ conditional on all possible realizations of its predecessors *X*^1^,..,*X*^*s*−1^, we can consider only its set of parents *Pa*(*X*^*s*^). More precisely, a set of variables *Pa*(*X*^*s*^) is defined as the Markovian parents of *X*^*s*^ if *Pa*(*X*^*s*^) is a minimal set of predecessors of *X*^*s*^ that makes *X*^*s*^ independent on all the other predecessors.

The joint probability distribution can therefore defined as:

(30) $$\begin{array}{@{}rcl@{}} P\left({x_{i}^{1}},..,{x_{i}^{m}}\right)=\prod\limits_{s}P\left({x_{i}^{s}}|Pa\left({x_{i}^{s}}\right)\right) \end{array} $$

where $P\left ({x_{i}^{s}}|Pa\left ({x_{i}^{s}}\right)\right)$ is described by a conditional probability distribution (CPD). These local conditional distributions correspond to the set *θ* of parameters.

**Figure 2:**
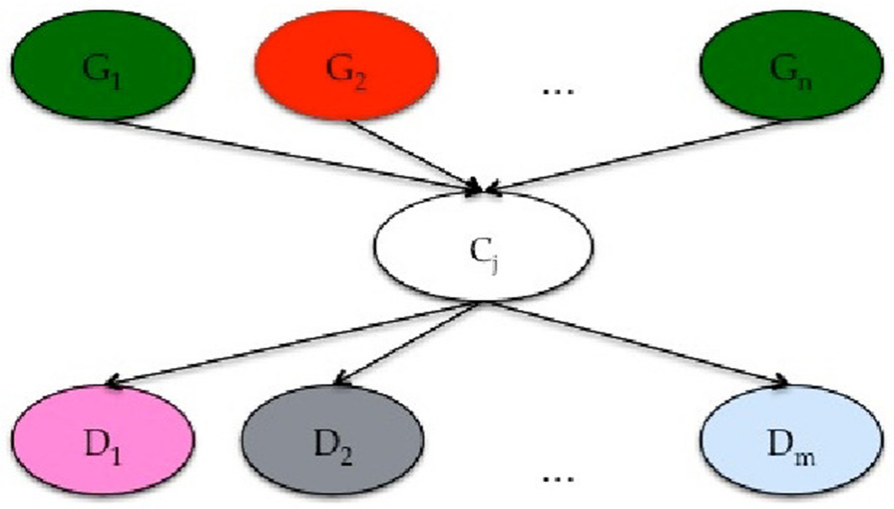
**NCI60 Bayesian network.** The upper part of the network comprises the top ten genes selected either by the IG or CFS feature selection, the central node corresponds the the cluster variable and the bottom nodes correspond to the drug responses to be predicted.

Figure 2 shows the dependency structure of BN used for the prediction task. We could gain an insight of how the expression pattern of genes influences the activity level of drugs through the cluster assignment. This structure of BN has been defined to train a probabilistic model able to simultaneously predict the drug responses of a new cell line, only by providing its (selection of) gene expression profile^c^.

The upper part of the network, which comprises 10 nodes, represents the most relevant genes selected by the policies described in the previous sections. The central part of the network, which is composed of only one variable, denotes the cluster obtained by solving the clustering problems. The bottom part, which comprises *n*=1400 nodes, represents the drug responses to be predicted. These last variables have been discretized in order to train discrete CPDs and consequently a fully discrete BN. In particular, following the discretization introduced in [33], cell lines with log10(GI50) at least 0.8 SDs above the mean were defined as resistant to the compound, whereas those with log10(GI50) at least 0.8 Standard Deviations below the mean were defined as sensitive. Cell lines with log10(GI50) within 0.8 Standard Deviations of the mean were considered to be intermediate. The remaining cell lines within 0.8 Standard Deviations were defined as intermediate. After this discretization process, the CPDs related to the dependency structure of the BN can be easily estimated, to then simultaneously predict the response of *n* drugs given the expression value of the 10 relevant genes.

## Results and discussion

In order to evaluate the quality of the proposed framework, a three-fold analysis has been performed. In our experimental investigation we consider *K*=9 clusters to be obtained, which respects the number of tumor types considered by the NCI60 panel. In order to avoid overfitting and to report unbiased experiments, each step of the proposed framework (clustering, feature selection, discretization and prediction) has been enclosed in a leave-one-out cross validation. For seek of clarity, the leave-one-out procedure works as follows:

- a cell line *x*_*i*_ is removed from the training set *Ω*
- clustering, feature selection, discretization and BN training are performed on the set {*Ω*∖*x*_*i*_}
- the removed cell line *x*_*i*_ is then used as test for prediction in BN

Results reported in the following are therefore averaged over the leave-one-out folds. The first analysis is concerned with the average Pearson Correlation Coefficient for estimating how homogeneous the clusters are. Given the obtained *K* clusters, the Pearson Correlation Coefficient *R* is computed as follows:

(31) $$ R=\sum\limits_{k=1}^{K}\frac{n_{k}}{|\Omega|} \left[\frac{2}{n_{k}\left(n_{k}-1\right)}\sum\limits_{i<j}corr\left(x_{i},x_{j}\right)z_{ik}z_{jk}\right]  $$

where *n*_*k*_ is the cardinality of cluster *k*. More specifically, the coefficient *R* has been computed with respect both to the gene and to the drug space, originating then two correlation coefficients: *R*^*G*^ is computed considering the correlation between instances represented by their gene expression profiles, while *R*^*D*^ is estimated considering the correlation between instances represented by their drug response profiles.

We also report the correlation indices of some baseline clustering approaches previously investigated for mining the NCI60 dataset: k-Means [14], Soft Topographic Vector Quantization (SVTQ) [9] and Relational k-Means [11].

**Figure 3:**
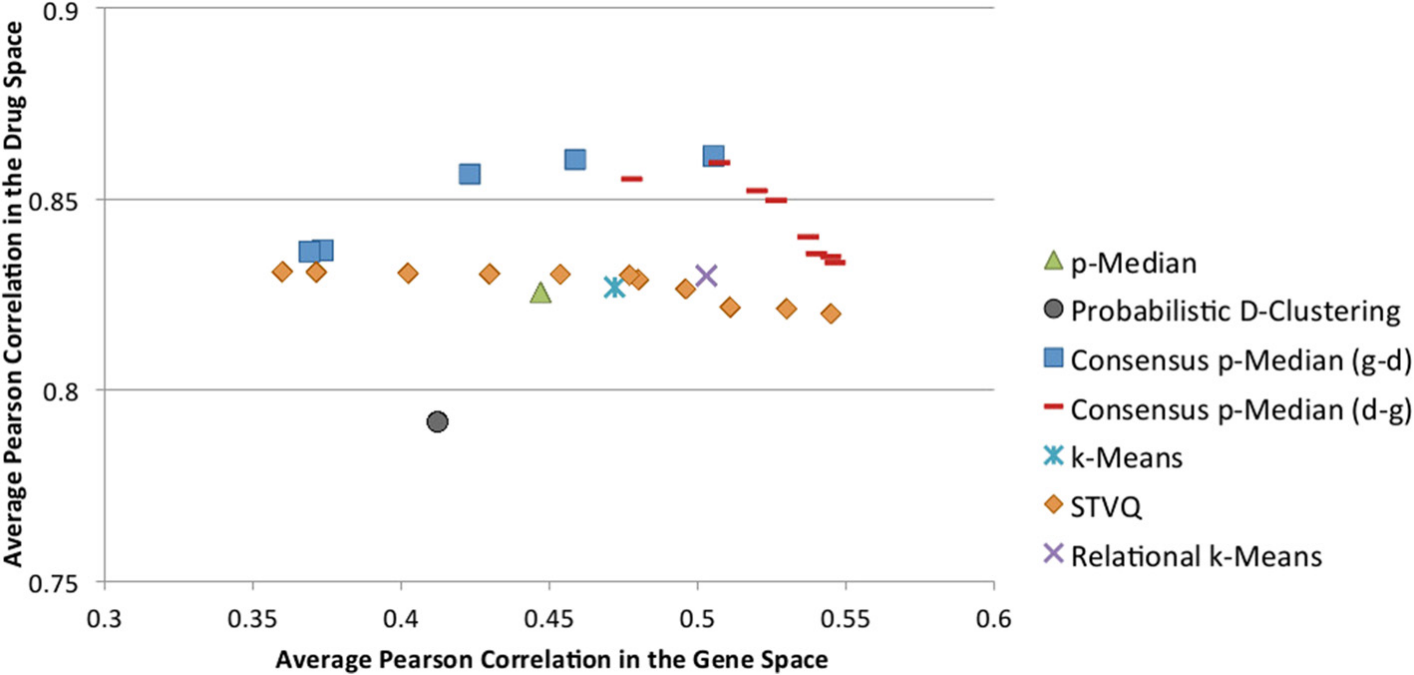
**Correlation indices for the Sherf dataset.** The y and x-coordinates denote the average Pearson correlation in the drug and gene space respectively. The correlation indices for all the reported series have been averaged over the leave-one-out cross validation folds. Each point of the series for Consensus p-Median corresponds to a solution obtained according to the parameter *μ*, while the series for STVQ reports values for *α*={0,0.1,0.2,⋯,1.0}.

**Figure 4:**
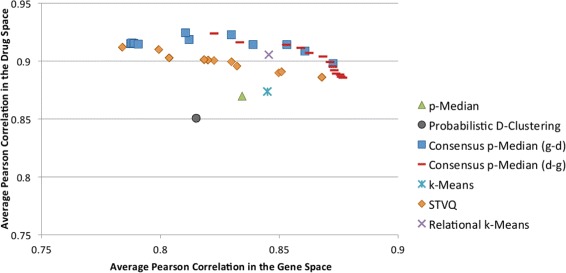
**Correlation Indices for the Liu dataset.** The y and x-coordinates denote the average Pearson correlation in the drug and gene (miRNA) space respectively. The correlation indices for all the reported series have been averaged over the leave-one-out cross validation folds. Each point of the series of Consensus p-Median corresponds to a solution obtained according to the parameter *μ*, while the series for STVQ reports values for *α*={0,0.1,0.2,⋯,1.0}.

In Figures 3 and 4, a comparison in terms of correlation (averaged on the leave-one-out folds) between the investigated clustering approaches is depicted reporting the traditional p-Median, Probabilistic D-Clustering, k-Means, SVTQ, Relational k-Means and the proposed Consensus p-Median. For the Consensus p-Median two series are reported, i.e. Consensus p-Median (g-d) and Consensus p-Median (d-g).

Each point of the series for the Consensus p-Median corresponds to a solution obtained according to the parameter *μ*. The ordinate axis represents the correlation coefficients in the drug space (*R*^*D*^ values), while the abscissae axis the correlation in the gene space (*R*^*G*^ values).

An interesting remark is related to the average correlation indices of the proposed approach. All the solutions provided by the Consensus p-Median show a slightly better (averaged) Pearson Coefficient than the others. This implies that our approach leads to clusters that are more homogeneous both in terms of gene expression and drug activity than the clusters obtained by the other approaches. This is highlighted by the fact that most of the solutions determined by the Consensus p-Median dominate the ones generated by the other approaches. The most promising “competitor” is Relational k-Means, which leads to almost homogeneous cluster configuration. In order to validate the significance of the results, confidence intervals have been estimated on the clustering solutions. Confidence intervals provide a range about the observed “effect size”, allowing us to understand how likely the generated solutions are: the smaller the confidence interval, the more certain we are about the solution. In our specific case, the confidence intervals have been computed as follows. First, for each run *l* of the leave-one-out, the Pearson Correlation Coefficients *R*_*l*_ (specifically ${R^{G}_{l}}$ and ${R^{D}_{l}}$) have been estimated. Then, the mean and the confidence interval have been estimated over the leave-one-out results.

In Table 1 confidence intervals are reported for the investigated clustering approaches on the Sherf dataset. We can easily note that the leave-one-out cross validation procedure provides a small effect size for most of the approaches both for correlations in the gene and drug space. We can state therefore that, with a confidence level of 95%, that the results are robust, enabling an easy identification of optimal solutions as Pareto points (marked as bold). Similar results have been obtained on the Liu dataset.

**Table 1:** **Confidence interval (at the 95%) level of the clustering solutions on the Sherf dataset**

		**Gene correlation**		**Drug correlation**	
		${\widehat {R}^{G}}$	**Confidence** ***±***	${\widehat {R}^{D}}$	**Confidence** ***±***
	*μ*=1.1	0.4777	0.0015	0.8553	0.0007
	*μ*=1.2	**0.5073**	0.0014	**0.8595**	0.0010
	*μ*=1.3	**0.5200**	0.0015	**0.8522**	0.0009
	*μ*=1.4	**0.5265**	0.0012	**0.8497**	0.0006
Consensus p-Median (d-g)	*μ*=1.5	**0.5373**	0.0008	**0.8401**	0.0005
	*μ*=1.6	**0.5401**	0.0013	**0.8357**	0.0010
	*μ*=1.7	**0.5449**	0.0008	**0.8349**	0.0006
	*μ*=1.8	0.5464	0.0011	**0.8334**	0.0007
	*μ*=1.1	**0.5054**	0.0010	**0.8613**	0.0008
	*μ*=1.2	0.4586	0.0009	0.8604	0.0006
Consensus p-Median (g-d)	*μ*=1.3	0.4232	0.0016	0.8566	0.0014
	*μ*=1.4	0.3735	0.0012	0.8366	0.0008
	*μ*=1.5	0.3689	0.0011	0.8363	0.0007
	*α*=0.0	0.5450	0.0037	0.8200	0.0035
	*α*=0.1	0.5300	0.0031	0.8213	0.0030
	*α*=0.2	0.5110	0.0034	0.8217	0.0019
	*α*=0.3	0.4960	0.0045	0.8265	0.0033
	*α*=0.4	0.4800	0.0039	0.8289	0.0042
STVQ	*α*=0.5	0.4770	0.0058	0.8301	0.0028
	*α*=0.6	0.4536	0.0051	0.8303	0.0031
	*α*=0.7	0.4298	0.0031	0.8304	0.0039
	*α*=0.8	0.4022	0.0029	0.8306	0.0033
	*α*=0.9	0.3713	0.0046	0.8309	0.0027
	*α*=1.0	0.3598	0.0028	0.8310	0.0029
p-Median	−	0.4596	0.0015	0.8366	0.0008
k-Means	−	0.4770	0.0058	0.8301	0.0028
Relational k-Means	−	0.4983	0.0023	0.8240	0.0012
Probabilistic D-Clustering	−	0.4122	0.0236	0.7916	0.0160

Pareto points have been marked as bold.

Concerning the computational complexity related to p-Median problems, it is well known that they belong to the NP-Hard complexity class. However, some recent meta-heuristcs allow to solve the p-Median problems in $\mathcal {O}\left (|\Omega |^{2}\right)$ making these kind of approaches competitive in respect of others. While a p-Median can be solved in $\mathcal {O}\left (|\Omega |^{2}\right)$, approaches like k-Means, Relational k-Means and STVQ have a computational complexity of $\mathcal {O}\left (|\Omega |IKQ\right)$ (where *Q* is related to the time spent on computing vector distances during the iterative procedure, *I* denotes the fixed number of iterations and *K* the clusters to be obtained). Considering that in our case *Q*≫|*Ω*| because *Q* depends on the vector dimension $\mathbb {R}^{m+n}$, it follows that a p-Median approach is more efficient than others.

**Figure 5:**
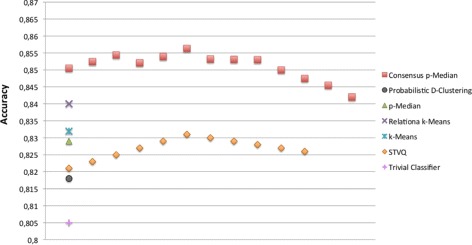
**Comparison of BNs accuracy on Sherf dataset.** The BNs have been trained according to the selection of genes by means of IG policy.

**Figure 6:**
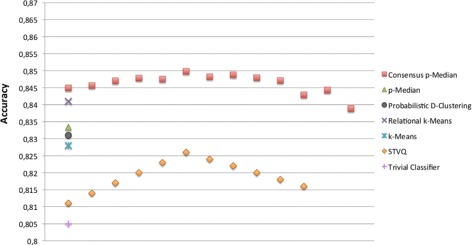
**Comparison of BNs accuracy on Sherf dataset.** The BNs have been trained according to the selection of genes by means of CFS policy.

**Figure 7:**
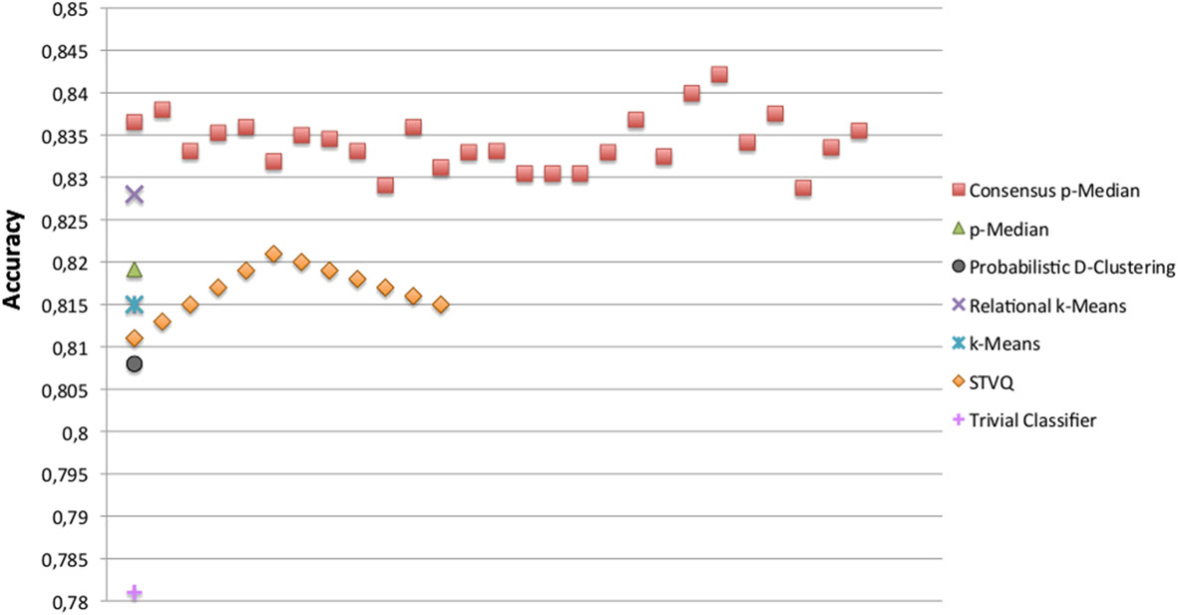
**Comparison of BNs accuracy on Liu dataset.** The BNs have been trained according to the selection of genes by means of IG policy.

**Figure 8:**
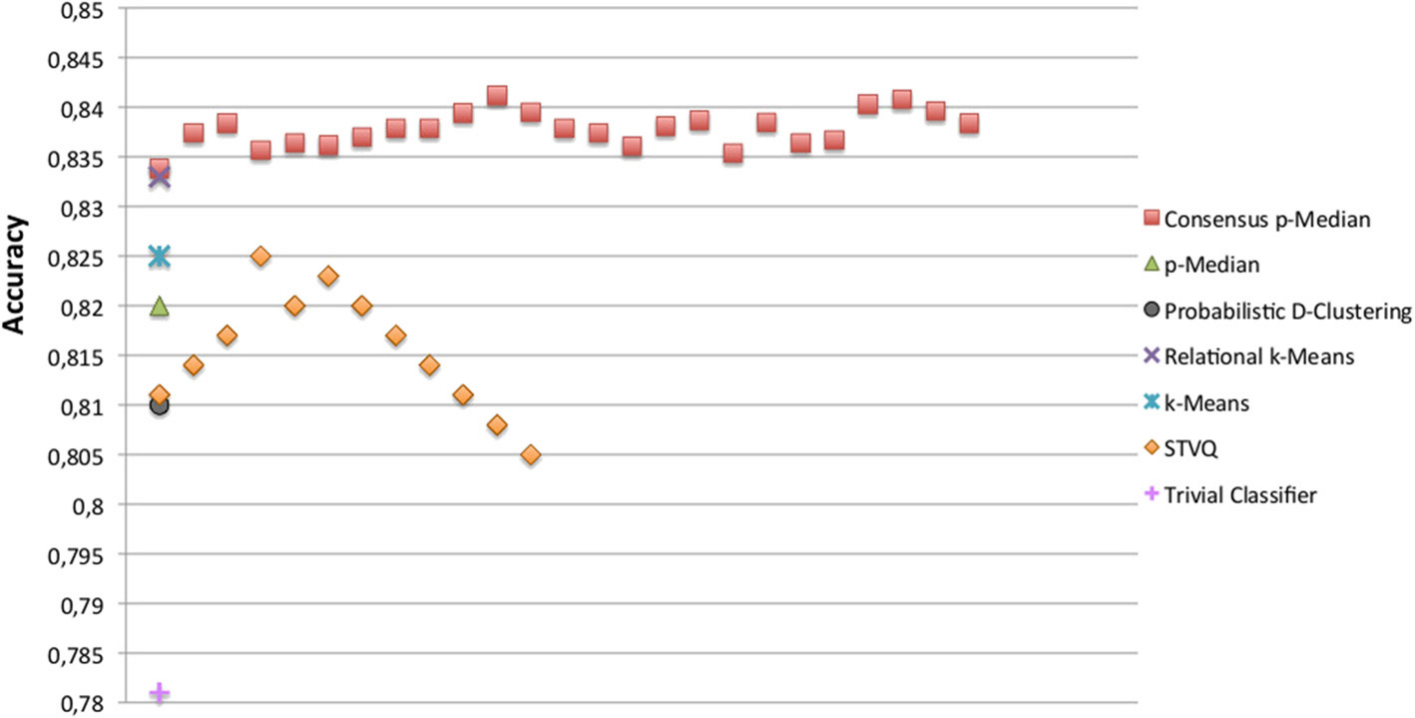
**Comparison of BNs accuracy on Liu dataset.** The BNs have been trained according to the selection of genes by means of CFS policy.

A further validation is targeted at the correctness of Bayesian Networks to predict the drug responses. In particular, we have measured the prediction accuracy of BNs trained with the top relevant genes characterizing the groups of cell lines derived by the mentioned clustering approaches. We have also reported the accuracy of a trivial classifier as baseline, where the prediction of a drug response is performed according to its majority class on the training data. In Figures 5, 6, 7 and 8 the comparison in terms of accuracy, i.e. percentage of drug response correctly predicted, between the investigated approaches is shown. The BNs are trained according to the (top ten) genes selected by the Information Gain and Correlation-based Subset Evaluation policies. In particular, considering that the experimental investigation is performed by means of a leave-one-out cross validation, the relevant genes to be used for training BNs have been selected as the most frequent over the top ten genes selected for each fold of the cross validation. Specifically, given the *L*=60 solutions obtained by performing a leave-one-out (for each given clustering approach), a voting mechanism has been applied. Each gene received a vote if, in a given run of the leave-one-out, it appears in the top ten list of relevant genes. Once the votes have been collected, the 10 genes with the highest number of votes are selected as the most important and therefore used to train the BN. It can be easily noted that all the solutions generated by the proposed approach outperform the ones obtained by the other methods. Concerning the Sherf dataset (Figures 5 and 6), the BNs trained according to the Consensus p-Median are able to ensure an average prediction accuracy of 85.63% with IG and 84.98% CFS selection policies, outperforming the accuracy of Probabilistic D-Clustering (81.8% with IG and 83.1% with CFS), p-Median (82.9% with IG and 83.33% with CFS), STVQ (83.1% with IG and 82.6% with CFS), k-Means (83.2% with IG and 82.8 with CFS), Relational k-Means (84.0% with IG and 84.2 with CFS) and the trivial classifier (80.5%).

Confidence intervals reported in Table 2 show not only the ability of IG selection policy to obtain small variability in the expected predictions, but also the correspondence between Pareto points and the most promising (in terms of average accuracy) Bayesian Networks (analogous results have been obtained on Liu dataset). This correspondence allows us to assert that the proposed Consensus p-Median is able to create groups of homogeneous cell lines taking into account two different data source and, as consequence, to derive a prediction model that outperforms the others. A similar result is obtained on the Liu dataset (Figures 7 and 8), where the proposed approach achieves more accurate predictions with respect to the other ones.

**Table 2:** **Confidence interval (at the 95%) level of the BN predictions (accuracy) on the Sherf dataset**

		**IG**		**CFS**	
		**Average**	**Confidence** ***±***	**Average**	**Confidence** ***±***
	*μ*=1.1	85.05	0.50	84.49	0.59
	*μ*=1.2	**85.24**	0.53	**84.56**	0.60
	*μ*=1.3	**85.44**	0.55	**84.70**	0.57
	*μ*=1.4	**85.21**	0.57	**84.78**	0.56
Consensus p-Median (d-g)	*μ*=1.5	**85.39**	0.59	**84.75**	0.64
	*μ*=1.6	**85.63**	0.53	**84.98**	0.60
	*μ*=1.7	**85.32**	0.54	**84.82**	0.59
	*μ*=1.8	**85.31**	0.55	**84.88**	0.56
	*μ*=1.1	85.30	0.52	**84.79**	0.60
	*μ*=1.2	85.00	0.44	84.71	0.55
Consensus p-Median (g-d)	*μ*=1.3	84.75	0.48	84.29	0.69
	*μ*=1.4	84.55	0.55	84.43	0.63
	*μ*=1.5	84.20	0.52	83.88	0.60
	*α*=0.0	82.10	0.47	81.10	0.49
	*α*=0.1	82.30	0.49	81.40	0.54
	*α*=0.2	82.50	0.53	81.70	0.56
	*α*=0.3	82.70	0.51	82.00	0.59
	*α*=0.4	82.90	0.55	82.30	0.60
STVQ	*α*=0.5	83.10	0.55	82.60	0.61
	*α*=0.6	83.00	0.58	82.40	0.61
	*α*=0.7	82.90	0.49	82.20	0.53
	*α*=0.8	82.80	0.45	82.00	0.50
	*α*=0.9	82.70	0.42	81.80	0.52
	*α*=1.0	82.60	0.41	81.60	0.51
p-Median	−	82.90	0.49	83.34	0.61
k-Means	−	83.10	0.59	82.80	0.65
Relational k-Means	−	84.00	0.56	84.10	0.60
Probabilistic D-Clustering	−	81.80	1.00	83.10	0.77

Predictions corresponding to Pareto points have been marked as bold.

**Figure 9:**
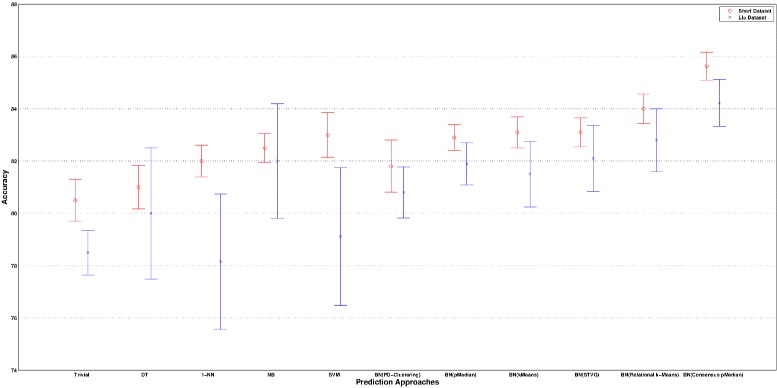
**Comparison of confidence intervals on prediction accuracy.** Results for Sherf and Liu dataset are reported (red and blue series respectively). Accuracy of traditional classifiers, i.e. DT, 1-NN, NB and SVM, are based on train/infer one drug at a time by using all the available genes. Results corresponding to the clustering approaches are concerned with BNs trained according to the clustering output and the IG feature selection policy.

Considering that the ultimate goal of this paper is the prediction of anticancer drug responses, we have also compared the BNs trained according to the Consensus p-Median with some traditional supervised methods. In particular, our approach has been compared with Naive Bayes (NB) [34], Decision Tree (DT) [35], 1-Nearest Neighbor (1-NN) [36] and Linear Support Vector Machines (SVM) [37] classifiers. Each model has been trained to predict one drug at a time by using all the available genes. Also for these classifiers a leave-one-out validation has been performed. For the Sherf Dataset, our approach obtained the highest performance with 85.63% of accuracy, against the ones of DT (81.00%), 1-NN (82.00%), NB (82.51%) and SVM (83.01%). Concerning the Liu dataset, similar results have been obtained. In particular, the proposed approach is able to achieve an accuracy of 84.22% compared with DT (80.00%), 1-NN (78.16%), NB (82.00%) and SVM (79.11%). A summary of the accuracy confidence intervals, both for Sherf and Liu datasets, is depicted in Figure 9. We can easily point out that the models based on RNA (Sherf dataset) are able to achieve higher average accuracy, with smaller confidence intervals, than the models based on microRNA (Liu dataset). A more interesting remark relates to the comparison of the considered models. Figure 9 confirms that the proposed approach, based on Consensus pMedian, guarantees not only the highest average prediction accuracy of drug response, but also a non-overlapping confidence interval. It’s also interesting to note that the performance of all the trained models have a small gap with respect to the trivial classifier, highlighting that (as similarly demonstrated in [38]) a relatively small number of drugs can be actually predicted better than the trivial baseline.

The last evaluation has been performed from a biological point of view in order to highlight the functional role of the most informative genes characterizing each cluster. Indeed, since clusters represent sets of cell lines that show a similar response to anti-cancer therapies also taking into account genomic information, the feature selection activity should be able to identify the subset of genes that could possibly regulate the cells response behaviour. In order to validate this hypothesis we searched for the biological functions associated to the selected genes by accessing the Entrez Gene Database ([39]), which is a NCBI’s (National Center for Biotechnology Information) database for gene-specific information ([40]). In Tables 3 and 4 we report the 10 genes that have been used to train the best Bayesian Network for the Sherf dataset, that correspond to the configuration obtained by the Consensus p-Median (d-g) with *μ*=1.6 both for IG and CFS. Column one reports the official gene name on Entrez Gene, column two contains a description of the main biological processes in which the gene is involved, and finally column three reports a description - extracted from the literature - of the role of the gene with respect to cancer mechanisms. Concerning IG selection policy on the Sherf dataset (Table 3), it’s interesting to highlight that most of the selected genes are recognized in the literature as biologically relevant. It’s also interesting to underline that some of the selected genes have been identified as relevant in previous investigations [7,11]: SPARC, SGK1, DNAJA3, ELF-3 and GJA1. A remarkable evidence is provided by genes CDKN2A and DNAJA3 as tumor suppressors, genes SPARC and GJA1 as tumor marker and finally SGK1 as prognostic marker. In particular SPARC, considered relevant in this study as well as in [7] and [11], has a reputation for being a potent anti-cancer. It has been shown to be involved in cell cycle, cell invasion, adhesion, migration, angiogenesis and apoptosis both in vitro and in vivo (see [41] for an overview of the multifunctional role of SPARC in cancer). Regarding CFS selection policy on the Sherf dataset (Table 4), we can note that some of the selected genes can be considered marginal from an oncological point of view, while others are shared with the ones obtained by applying IG (SPARC, DNAJA3 and AKT3).

**Table 3:** **Gene Selection (IG) based on Consensus p-Median on the Sherf dataset**

**Gene name**	**Biological process**	**Role (referred literature)**
SPARC	Regulation of cell proliferation; signal transduction	SPARC is a secreted protein, acidic and rich in cysteines. It is a matrix associated protein that elicits changes in cell shape, inhibits cell cycle progression, and influences the synthesis of extracellular matrix. Clinical evidence indicates that SPARC expression correlates with tumor progression [42]. The gene product has been associated with tumor suppression but has also been correlated with metastasis based on changes to cell shape which can promote tumor cell invasion [43].
MAP1B	Microtubule bundle formation; negative regulation of intracellular transport	MAP1B interacts with a wide variety of proteins, there is growing consideration that MAP1B plays a crucial role in cytoskeleton stability and may also have a role in other cellular functions as well [44]. DAPK-1 promotes autophagy by binding to the microtubule-associated protein MAP1B, which is an LC3 interactor with anti-autophagic functions [45].
DNAJA3	Apoptosis; cell death; negative regulation of cell proliferation	It is an important cell death regulator and could exert tumor suppressor activity [46]. The results establish DNAJA3 as a novel regulator of p53-mediated apoptosis, and suggest that therapies designed to enhance DNAJA3’s function in promoting mitochondrial localization of p53 and apoptosis could be an effective therapy in many cancers [47].
SGK1	Apoptosis	SGK1 is a downstream target of cell survival and that it is primarily regulated at the level of transcription [48,49].
ELF3	Inflammatory response;	Transcriptional inhibition of ELF3 could be a one of the mechanisms of colonic carcinogenesis [50].
CDKN2A	Cell cycle arrest; cell cycle checkpoint; negative regulation of cell growth; negative regulation of cell proliferation	CDKN2A is an important tumor suppressor gene and is specifically required for p53 activation under oncogenic stress [51]. Suppression of CDKN2A, a cell-cycle regulator, occurs in essentially all common human cancers [52]. Inactivating these tumor suppressors directly promotes tumorigenesis due to lack of control over cellular processes [53].
SPINT2	Cellular component movement	SPINT2 play important roles in controlling the aggressive nature and spread of cancer, displaying a unique therapeutic potential [54].
GJA1	Apoptosis	GJA1 is involved in several kinds of tumor, as breast, lung, prostate and ovarian [55,56] and [57].
AKT3	Signal transduction	AKT signaling pathway is activated in human cancers and consequences for molecularly targeted therapies. AKT isoform may play a positive or negative role in cell migration and invasion. AKT is also involved in regulation of tumor angiogenesis [58].
EpCAM	Positive regulation of cell proliferation	EpCAM has oncogenic potential and is activated by release of its intracellular domain, which can signal into the cell nucleus by engagement of elements of the wnt pathway [59]. Regulated intramembrane proteolysis activates EpCAM as a mitogenic signal transducer in vitro and in vivo [60].

The reported genes refer to the outperforming BN (85.63% of accuracy) trained according to the solution generated by the Consensus p-Median (d-g) with ***μ***=1.6.

**Table 4:** **Gene selection (CFS) based on consensus p-Median on the Sherf dataset**

**Gene name**	**Biological process**	**Role (referred literature)**
POLR2F	Protein kinase activity; DNA binding	POLR2F exhibited elevated levels in carcinomas compared to normal tissue samples suggesting a possible role for these molecules in colorectal cancer [61].
SPARC	Regulation of cell proliferation; signal transduction	SPARC is a secreted protein, acidic and rich in cysteines. It is a matrix associated protein that elicits changes in cell shape, inhibits cell cycle progression, and influences the synthesis of extracellular matrix. Clinical evidence indicates that SPARC expression correlates with tumor progression [42]. The gene product has been associated with tumor suppression but has also been correlated with metastasis based on changes to cell shape which can promote tumor cell invasion [43].
DNAJA3	Apoptosis; cell death; negative regulation of cell proliferation	It is an important cell death regulator and could exert tumor suppressor activity [46]. The results establish DNAJA3 as a novel regulator of p53-mediated apoptosis, and suggest that therapies designed to enhance DNAJA3’s function in promoting mitochondrial localization of p53 and apoptosis could be an effective therapy in many cancers [47].
PTN	Regulation of cell proliferation and division	PTN is an angiogenic factor and has been found to be constitutively expressed in many human tumors of different cell types [62].
AIF-1	Regulation of muscle cell proliferation	AIF-1 can promote the growth of breast tumors via activating NF-kappaB signaling [63].
STMN4	Regulation of microtubule polymerization or depolymerization	-
PSAP	Lipid BINDING	PSAP is involved in prostate cancer invasion [64] and inhibits tumor metastasis via paracrine and endocrine stimulation of stromal p53 and Tsp-1 [65].
AKT3	Signal transduction	AKT signaling pathway is activated in human cancers and consequences for molecularly targeted therapies. AKT isoform may play a positive or negative role in cell migration and invasion. AKT is also involved in regulation of tumor angiogenesis [58].
FBXO7	Cell death; protein binding	-
P4HA2	L-ascorbic acid binding	Overexpression of PRDX4 and P4HA2 was significantly associated with lymphatic metastasis in oral cavity squamous cell carcinoma [66]. P4HA2 was upregulated in breast tumor cells compared with its adjacent normal tissues [67].

The reported genes refer to the outperforming BN (84.98% of accuracy) trained according to the solution generated by the Consensus p-Median (d-g) with ***μ***=1.6.

Concerning the Liu dataset, the analysis of the selected relevant miRNAs can be only preliminary. Although miRNAs represent a recently discovered class of non-coding RNAs that play a fundamental role in the regulation of gene expression, most of their functions still remain to be discovered. For this reason, we can only report the genes whose mRNA can interact with the considered miRNA (see Table 5). The listed genes have been selected by accessing the data available at “microRNA.org - Targets and Expression” ([68]), which is a freely available open-source software able to provide microRNA target predictions [69]. In order to provide a preliminary evaluation of the selected miRNA, we can point out their involvement in the “microRNA in cancer” pathway (from KEGG source record *hsa05206*). As highlighted in Table 5, some miRNAs belong to the above mentioned pathway: hsa-miR-200a, hsa-miR-200b, hsa-miR-200c, hsa-miR-141, hsa-miR-100 and hsa-miR-494 identified by the IG selection, and hsa-miR-145 and hsa-miR-17* determined by the CFS policy. We highlight that (as for Sherf dataset) some miRNA are shared between the two selection policies (hsa-miR-196b, hsa-miR-18b and hsa-miR-100).

**Table 5:** **miRNA Selection (IG and CFS) based on Consensus p-Median on the Liu dataset**

**IG**		**CFS**	
**miRNA**	**Target gene**	**miRNA**	**Target gene**
**hsa-miR-200a**	AP3S1	hsa-miR-196b	HOXA7
hsa-miR-429	ZEB2	hsa-miR-18b	OTX2
**hsa-miR-200b**	ZEB2	hsa-miR-142–5p	SGPP1
**hsa-miR-200c**	ZEB2	hsa-miR-100	TMPRSS13
**hsa-miR-141**	AP3S1	hsa-miR-106a	DYNC1LI2
hsa-miR-196b	HOXA7	**hsa-miR-145**	FAM108C1
hsa-miR-18b	OTX2	hsa-miR-17*	HMGA2
**hsa-miR-100**	TMPRSS13	hsa-miR-376c	PAX4
hsa-miR-365	ZNF680	hsa-miR-211	ACSM2A
**hsa-miR-494**	ARID4B	hsa-miR-503	MYH10

The reported miRNAs refer to the outperforming BNs (84.22% of accuracy with IG and 84.12% with CFS): for IG the optimal BN is denoted by Consensus p-Median (g-d) with ***μ***=1.6, while for CFS the optimal BN is obtained by means of Consensus p-Median (d-g) with ***μ***=2.1. miRNAs belonging the “microRNA in cancer” pathway are marked as bold.

In order to analyze the computational time of the entire framework, a comparison over the considered datasets has been reported in Tables 6 and 7. The execution time (in terms of seconds) has been reported for the three phases, i.e. clustering, feature selection and prediction. Concerning the clustering phase, it can be noted in Tables 6(a) and 7(a) that the most efficient approach is traditional p-Median, followed by the proposed Consensus p-Median (the proposed approach has to solve two p-Median problems instead of only only one). Tables 6(b) and 7(b) report the computational effort required by the feature selection policies (IG and CFS) and training and inference phases needed for prediction with BNs. The selection strategy based on CFS is clearly computationally intensive because it requires the search of the sub-optimal set of features that could compactly represent the clusters. On the contrary, IG is more efficient thanks to its ability to evaluate each feature (gene) independently on the others. Considering BNs, the training step requires a quite limited computational effort because only Conditional Probability Tables need to be estimated (the dependency structure is fixed a priori). The time required by the inference step is mainly influenced by the number of drugs that are simultaneously predicted, i.e. more therapeutic compounds are considered and more time is necessary to estimate their posterior probability of being sensitive, resistant and intermediate.

**Table 6:** **Efficiency comparison (in terms of seconds) ofthe entire framework on Sherf dataset**

**(a) Clustering**	
**Clustering**	**Execution time**
Consensus p-Median (d-g)	0.504
Consensus p-Median (g-d)	0.425
STVQ	0.693
p-Median	0.250
k-Means	0.533
Relational k-Means	0.966
Probabilistic D-Clustering	0.590
**(b) Feature selection and prediction**	
**Feature selection**	**Execution time**
IG	0.27
CFS	3980
**Prediction**	**Execution time**
Training	9.68
Inference	110.227

**Table 7:** **Efficiency comparison (in terms of seconds) ofthe entire framework on Liu Dataset**

**(a) Clustering**	
**Clustering**	**Execution time**
Consensus p-Median (d-g)	0.398
Consensus p-Median (g-d)	0.381
STVQ	0.473
p-Median	0.215
k-Means	0.446
Relational k-Means	0.687
Probabilistic D-Clustering	0.482
**(b) Feature selection and prediction**	
**Feature selection**	**Execution time**
IG	0.130
CFS	1321
**Prediction**	**Execution time**
Training	0.604
Inference	4.791

As final remark, considering both qualitative and quantitative results, we can assert that Consensus p-Median together with Information Gain and Bayesian Network represent an optimal trade-off between efficacy and efficiency to simultaneously predict (in silico) anticancer responses.

## Conclusion

In this paper the problem of identifying a suitable profile of cancer patients by linking gene expressions, drug responses and types of cancer has been addressed. A learning framework based on three building blocks has been proposed. The experimental results highlight three main findings: (1) the proposed Consensus p-Median is able to create groups of cell lines that are highly correlated both in terms of gene expression and drug response; (2) from a biological point of view, the gene selection performed on these clusters allows the identification of genes that are strongly involved in several cancer processes; (3) the prediction of drug responses, by using the patient profile obtained through clustering and gene selection, represents a promising step for predicting potentially useful drugs. Concerning the ongoing research, several issues are still to be investigated. Among them the next future work will be focused to the identification of a suitable number of clusters and the use of more “selective” discretization policies. As far is concerned with the methodological approach, an interesting comparison relates with those approaches, belonging to the multiple tasks learning, able to simultaneously predict the drug responses given a (subset) of gene expressions. Instances of future investigations are Marginal Regression [70] and Support Vector Machine [71] For Multitask Learning. A further development of the proposed investigation relates to the exploitation of additional data sources, such as proteomic expression profiles, to better predict the drug response in tumour cells.

## Availability of supporting data

The data sets supporting the results of this article are included within the article (and its additional files).

## Endnotes

^a^ The solutions of the p-Median problems have been determined by using the CPLEX commercial solver.

^b^ For the feature selection process, the WEKA environment [72] has been exploited [73].

^c^ For training and inference of Bayesian Networks, the BNT Matlab toolbox has been used. The toolbox is available for download at [74].

## Additional files

Additional file 1
**Sherf gene expression data.** Gene expression profiles of NCI-60 cell lines: 1375 genes for 60 cell lines.

Additional file 2
**Sherf drug activity data.** Drug activity patterns of NCI-60 cell lines: 1400 compounds for 60 cell lines.

Additional file 3
**Liu miRNA expression data.** miRNA expression profiles of NCI-60 cell lines: 422 miRNA for 60 cell lines.

Additional file 4
**Liu drug activity data.** Drug activity patterns of NCI-60 cell lines: 118 drugs in clinical use for 60 cell lines.
